# An innovative approach to providing lifestyle education and behaviour change to prevent type 2 diabetes

**Published:** 2012-06-15

**Authors:** Katherine Grady, Linda Savas

**Affiliations:** Salford Royal Foundation Trust, UK; Greater Manchester Collaboration for Leadership in Applied Health Research and Care (CLAHRC), UK

**Keywords:** diabetes prevention, telephone, lifestyle intervention

## Abstract

**Introduction:**

Diabetes is one of the major health challenges of our time. Diabetes UK recently estimated 10% of the total NHS budget is spent on diabetes care. NICE guidance “Prevention of type 2 diabetes in adults” (2011) and “Prevention of type 2 diabetes in high-risk groups” (currently consultation phase) emphasises the importance of prevention. Impaired glucose tolerance (IGT) is a precursor for the development of type 2 diabetes and is additionally associated with increased cardiovascular risk. Positive lifestyle changes (healthy eating, increased activity, weight reduction) have been proven to prevent or delay onset of type 2 diabetes in people diagnosed with IGT.

**Aims and objectives:**

Working together, Greater Manchester CLAHRC and Salford’s NHS Diabetes Care Call team developed a six-month, telephone-based, lifestyle intervention programme for people with IGT. The aim was to provide a convenient, accessible and tailored service that would motivate and enable people to make positive behaviour changes to prevent or delay onset of type 2 diabetes. The programme was delivered by a team of trained health advisors who provided standardised, evidence-based education via a series of electronic scripts developed and maintained by the specialist diabetes team. Supporting resources, including a patient education leaflet and DVD designed in-house, were sent by post. Health advisors worked on an individual basis with participants and had access to an online directory of local services and groups to signpost appropriately. The project ran from May 2010 to January 2011, enrolling 55 people with IGT from seven GP practices in Salford. All calls were recorded on the electronic patient record, viewable across primary and secondary care.

**Key results:**

All 55 participants completed the pathway.

**Clinical Outcomes:**

52% (n=26) reverted to normal fasting and glucose tolerance.

10% (n=5) reduced risk to impaired fasting glucose.

75% (n=38) confirmed weight loss, average 4.8 kg (5.3%) per person.

61% (n=31) reduced FINDRISC score by average 2.1 points per person.

88% (n=48) achieved or partially achieved their overall lifestyle goal.

**Qualitative outcomes (from questionnaires and focus groups):**

88% (n=36) participants reported increased understanding of blood results.

78% (n=32) participants reported definite increased confidence about how to reduce their own risk of developing type 2 diabetes.

90% (n=37) felt they received relevant, up to date advice about reducing diabetes risk.

GP practices reported high-levels of confidence that the service provided evidence-based dietary and lifestyle advice and motivational support.

Large-scale research studies strongly suggest lifestyle programmes preventing diabetes are cost effective. Cost benefit analysis of this project and extrapolation for whole population shows roll out is achievable with payback on investment in year three.

**Conclusion:**

The IGT care call service is a highly scalable and cost-effective approach to preventing diabetes and amenable for ‘hard to reach’ groups. A full evaluation report was presented to NHS Salford Commissioning Board who have since allocated further funding, allowing further expansion of the project and longer-term follow-up of participants. This project recently won a Quality in Care (QiC) Diabetes award for “Best type 2 diabetes prevention initiative” (November 2011).

